# Patients with Systemic Lupus Erythematosus Have Higher Prevalence of Thyroid Autoantibodies: A Systematic Review and Meta-Analysis

**DOI:** 10.1371/journal.pone.0123291

**Published:** 2015-04-23

**Authors:** Xi-Feng Pan, Jian-Qiu Gu, Zhong-Yan Shan

**Affiliations:** Department of Endocrinology and Metabolism, Institute of Endocrinology, Liaoning Provincial Key Laboratory of Endocrine Diseases, The First Affiliated Hospital, China Medical University, Shenyang, Liaoning Province, China; University of Texas Health Science Center at Houston, UNITED STATES

## Abstract

**Background:**

Thyroid autoimmunity is considered the most common type of organ-specific autoimmune disorder and can be associated with other autoimmune endocrine disorders or non-endocrine diseases. Systemic lupus erythematosus is a prototypical autoimmune disorder with multifactorial etiology. The pathogenesis and development of the disease may result from a loss of immune tolerance and the resulting synthesis of autoantibodies against nuclear antigens. Autoimmune factors may be common features of both thyroid autoimmunity and systemic lupus erythematosus, making it likely that both conditions may coexist within some patients.

**Methods and Findings:**

A number of studies have investigated whether an association between thyroid autoimmunity and systemic lupus erythematosus exists. However, the results of these studies have been inconsistent. Furthermore, most of these studies have had relatively small sample sizes, which have rendered them insufficiently powerful to determine whether there is an association between systemic lupus erythematosus and thyroid autoimmunity. The main objective of this meta-analysis is to provide reliable estimates of the extent of any association between thyroid autoimmunity and systemic lupus erythematosus by combining the primary data from all relevant studies. Literature databases were searched, including the Medline, Embase, Web of Science, Chinese Wanfang and CBM databases, from January 1970 to May 2014. A total of 1076 systemic lupus erythematosus cases and 1661 healthy controls were included in this study. From these data, the odds ratio (OR) and the corresponding 95% confidence interval (95% CI) were calculated. The meta-analysis results showed that the prevalence of thyroid autoantibody positivity in patients with systemic lupus erythematosus was higher than in healthy controls (TgAb: OR = 2.99, 95% CI = 1.83–4.89; TPOAb: OR = 2.20, 95% CI = 1.27–3.82, respectively).

**Conclusion:**

The results of this meta-analysis suggest that thyroid autoimmunity is more prevalent in patients with systemic lupus erythematosus than in a control group.

## Introduction

A lot of endocrine glands such as the thyroid, adrenal, and pancreatic islet beta cells are affected by autoimmune diseases. However, thyroid autoimmunity is one of the most common autoimmune endocrine diseases [1]. Graves’ disease and Hashimoto’s thyroiditis are the most common clinical expressions of this disorder. GD is characterized by hyperthyroidism because of excessive production of thyroid hormone caused by specific autoantibodies to thyrotropin receptor. However, HT is a T cell-mediated autoimmune disease that leads to clinical hypothyroidism because of thyroid destruction [2]. The character of thyroid autoimmunity is the production of thyroid autoantibodies. Nevertheless, the underlying mechanisms by which specific antibodies against thyroid tissue are produced are still unknown. Recent studies have illustrated that both endogenous and exogenous factors affect the severity of autoimmune reactions by inducing innate immune responses [3].

Systemic lupus erythematosus (SLE) is characterized by autoantibodies production, immune complex deposition, hyperactive T and B cells and multi-organ damage, which is a prototypical autoimmune disorder with strong genetic influence [4]. Autoantibodies are produced by activation of the immune system, ascribing to the loss of immune tolerance for self-antigens, which in turn result in the clinical manifestations of this disorder. The pathogenesis of SLE is considered to be influenced by many factors, such as genetic, hormonal and environmental factors [5]. Because autoimmunity plays an important role in the pathogenesis of SLE, an association between this disorder and thyroid autoimmunity may exist.

A number of studies have investigated the association between thyroid autoimmunity and SLE by assaying the prevalence of thyroid autoantibodies, including thyroglobulin antibody (TgAb) and thyroid peroxidase antibody (TPOAb). However, the results of these studies have been inconsistent. Furthermore, most of these studies examined a relatively small sample size and were thus not powerful enough to determine whether an association between SLE and thyroid autoimmunity exists. The main objective of this meta-analysis is to provide reliable estimates of the extent of any association between thyroid autoimmunity and SLE using combined primary data from all relevant studies.

## Materials and Methods

A completed PRISMA checklist is presented in the Supporting Information in S1 PRISMA Checklist.

### Literature and search strategy

A systematic search without language restriction was conducted for eligible studies listed from January 1970 through May 2014 in the Medline, Embase, Web of Science, Chinese Wanfang and CBM databases using the key words "systemic lupus erythematosus" or "SLE" in combination with the terms "thyroid autoimmunity" or "autoimmune thyroid diseases" or "autoimmune thyroiditis" or "hashimoto’s" or "grave’s" or "thyroid peroxidase antibody" or "thyroglobulin antibody", and the studies were filtered for those containing human subjects. In addition, the reference lists of the retrieved articles were reviewed to identify additional eligible studies. Studies were selected by two independent reviewers. The relevance of studies was assessed using a hierarchical approach on the basis of the title, abstract, and full manuscript. The studies selected by the two reviewers were compared, and disagreements were resolved by consensus.

The preliminary search using the above terms yielded 3074 potential articles. The authors read the abstracts of these 3074 publications and identified 689 publications that appeared to contain relevant data. These 689 publications were read in full and scrutinized for the presence of data. This process identified 15 studies appropriate for further analysis based on our inclusion criteria.

### Inclusion criteria

Eligible studies included in this meta-analysis met all of the following criteria: (1) evaluate the association between autoantibodies directed against thyroid tissue and SLE, (2) case-control design, (3) provide sufficient data of cases and healthy controls that could allow us to calculate the odds ratio (OR) with 95% confidence interval (CI) and a *P* value.

### Data extraction

The following information was extracted from each study: first author, publication year, geographical location of the study population, numbers of cases and controls, and numbers of cases and controls positive for each type of thyroid autoantibody. Information was carefully extracted from all eligible publications independently by two of the authors. Disagreement was resolved by discussion between the authors. If a consensus could not be reached, another investigator settled the disagreement.

### Assessment of risk of bias in included studies

Two investigators performed quality assessments using the Newcastle-Ottawa Scale for included studies [6]. The Newcastle-Ottawa Scale is a risk of bias assessment tool for observational studies that is recommended by the Cochrane Collaboration [7]. Each study included was judged in three broad categories by using the “star system”: the selection of study groups, the comparability of their cases and controls, and the ascertainment of exposure for cases and controls. When conforming to the criteria of an item, the study would get one star. The exception was the comparability category, for which a study could receive a maximum of two stars. The Newcastle-Ottawa Scale ranges between zero up to nine stars. As our meta-analysis focused on the relationship between SLE and thyroid autoimmunity, we explained the “no history of disease (endpoint)” item as “no history of SLE”. Furthermore, we created a funnel plot to analyze potential publication bias. The studies assessed by both investigators were compared, and disagreement was resolved by consensus.

### Statistical analysis

The association between thyroid autoimmunity and SLE was evaluated by OR with the corresponding 95% CI. The significance of OR was determined with a Z test (*P*<0.05 was considered statistically significant), and Cochrane’s *Q* test was performed to determine the extent of inter-study heterogeneity using a cutoff of *P*<0.1 as statistically significant. The OR, which was calculated using either a random effects model or a fixed effects model, was determined in accordance with the heterogeneity, as measured using Cochrane’s *Q* statistics. *I* ^*2*^ values of 25%, 50% and 75% were defined as low, moderate and high estimates, respectively. When a significant *Q* test (*P*<0.10) or *I* ^*2*^ >50% indicated heterogeneity across studies, the random effects model was used for the meta-analysis. Otherwise, the fixed effects model was adopted. The publication bias was examined by Begg’s test. All the statistical analyses were performed using *Stata Version 12*.*0* software.

## Results

### Characteristics of the included studies

**Fig 1:**
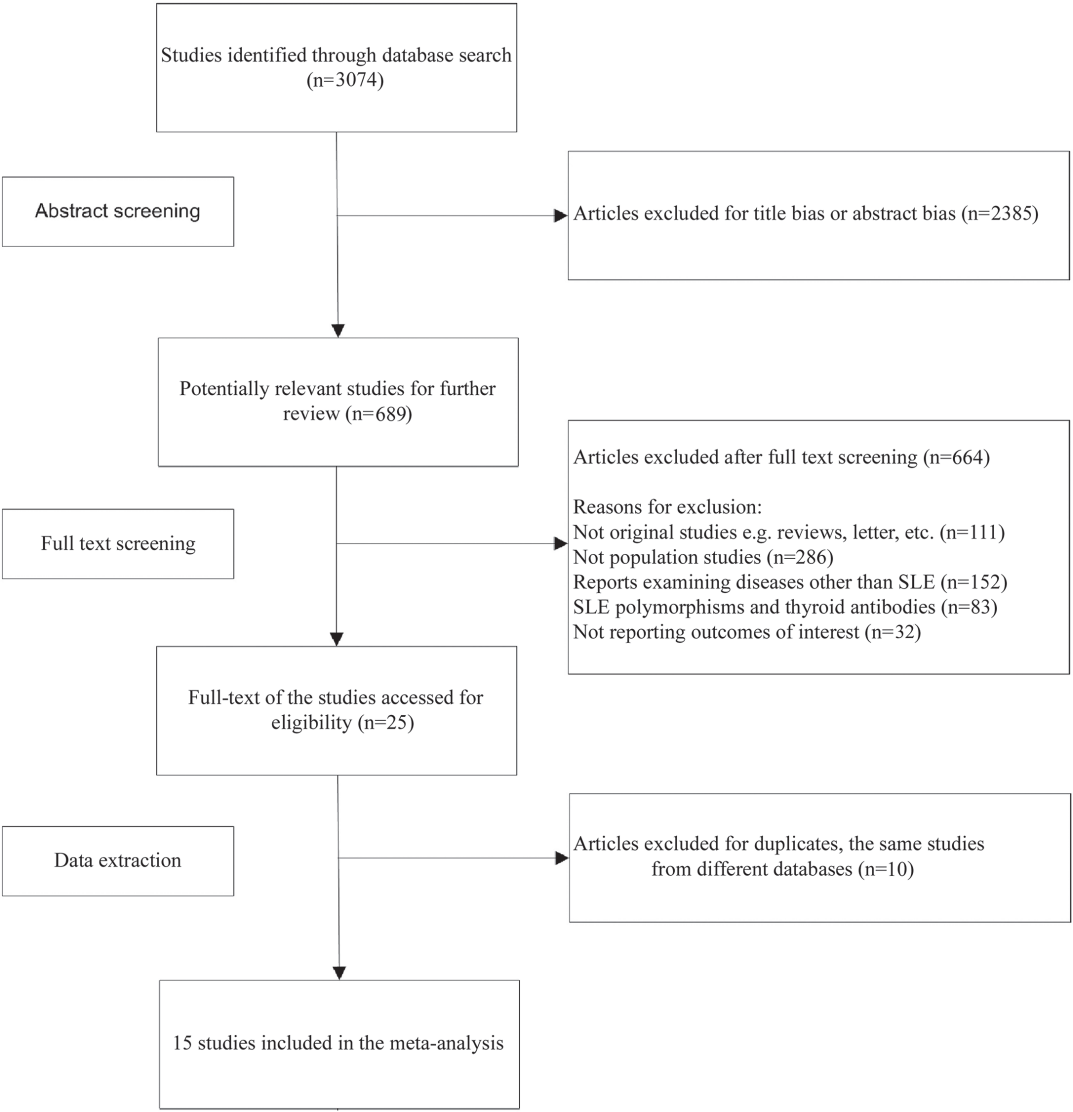
Flow charts show the detailed procedure for the inclusion or exclusion of studies. 15 independent articles were included in this meta-analysis.

A total of 15 relevant studies with a case-control design that investigated the relationship between thyroid autoimmunity and SLE by assaying for thyroid autoantibodies were identified that met the study inclusion criteria. The detailed procedure used to include or exclude studies is presented in Fig 1. We chose these eligible studies to perform our meta-analysis, and as a result, our meta-analysis included 1076 SLE cases and 1661 healthy controls. The characteristics of each study included in this meta-analysis are summarized in Table 1. The quality of the included studies was assessed according to the Newcastle-Ottawa Scale guidelines for case-control studies (S1 Table). Overall, in accordance with the suggested criteria for Selection, Comparability, and Exposure categories of the Newcastle-Ottawa Scale, the studies included in this meta-analysis were of acceptable quality.

**Table 1:** General characteristics of the studies included in this meta-analysis.

Authors. [Ref]	Year	Region	Continent	Thyroid antibodies	SLE cases (n / N)	Healthy controls (n / N)
Rivero et al. [8]	1974	Argentina	American	TgAb	19 / 93	4 / 100
Weetman et al. [9]	1987	UK	European	TgAb	15 / 41	9 / 41
Xu et al. [10]	1995	China	Asian	TgAb	9 / 42	0 / 35
El-Sherif et al. [11]	2004	Egypt	African	TgAb, TPOAb	1 / 20, 3 / 20	2 / 20, 2 / 20
Soukup et al. [12]	2004	Czech	European	TgAb, TPOAb	19 / 102, 16 / 102	4 / 59, 7 / 59
Kramer et al. [13]	2005	Brazil	American	TgAb, TPOAb	2 / 26, 3 / 26	0 / 28, 1 / 28
Kostić et al. [14]	2006	Serbia	European	TgAb, TPOAb	6 / 53, 12 / 53	1 / 34, 2 / 34
Mader et al. [15]	2007	Israel	Asian	TgAb, TPOAb	6 / 77, 4 / 77	4 / 52, 4 / 52
Al-Awadhi et al. [16]	2008	Kuwait	Asian	TgAb	7 / 60	6 / 577
Viggiano et al. [17]	2008	Brazil	American	TgAb, TPOAb	21 / 106, 16 / 106	8 / 102, 16 / 102
Assal et al. [18]	2009	Egypt	African	TgAb, TPOAb	2 / 30, 5 / 30	3 / 30, 3 / 30
Antonelli et al. [19]	2010	Italy	European	TgAb, TPOAb	33 / 213, 59 / 213	47 / 426, 53 / 426
Hrycek et al. [20]	2010	Poland	European	TgAb, TPOAb	3 / 41, 8 / 41	1 / 17, 4 / 17
Mousa et al. [21]	2012	Egypt	African	TgAb, TPOAb	11 / 132, 26 / 132	2 / 120, 7 / 120
El-saadany et al. [22]	2014	Egypt	African	TgAb, TPOAb	22 / 40, 34 / 40	2 / 20, 3 / 20

See S2 Table in the Supporting Information for additional details about the included studies. *TgAb*: thyroglobulin antibody. *TPOAb*: thyroid peroxidase antibody. *n*: number of individuals with positive antibodies. *N*: total number of individuals with SLE disease or healthy controls.

### Meta-analysis results

**Fig 2:**
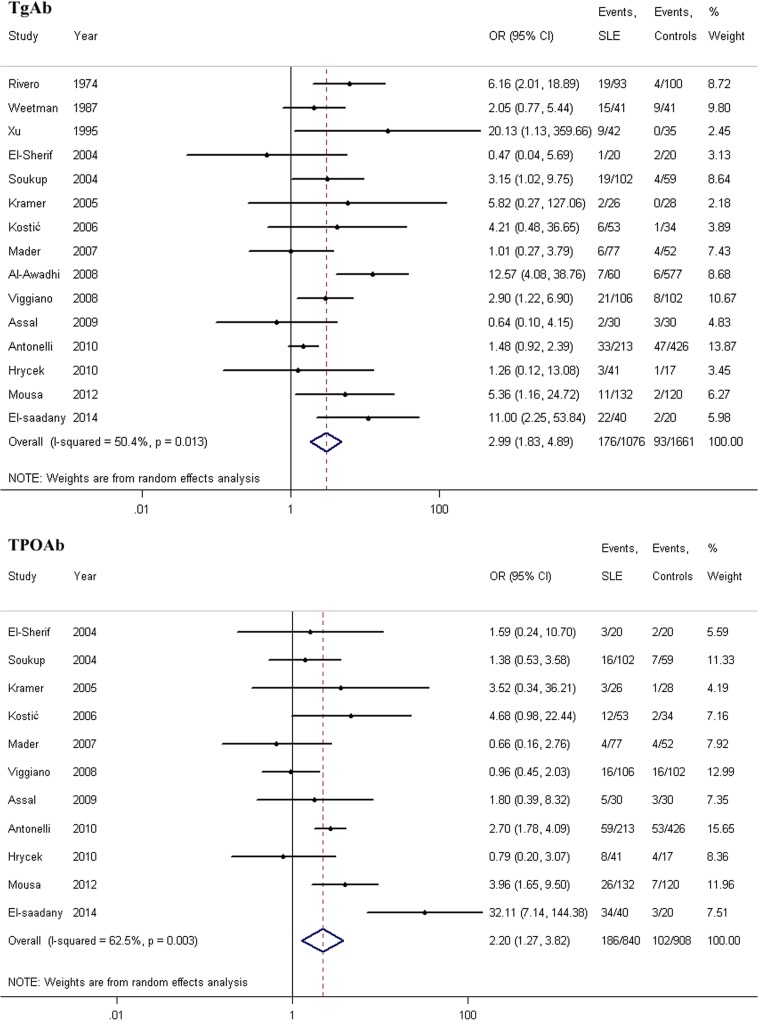
Forest plots for the prevalence of TgAb and TPOAb positivity in patients with SLE compared with healthy controls. The diamond represents the pooled OR and 95% CI.

A summary of the meta-analysis findings regarding the association between SLE and thyroid autoimmunity, after incorporating data from all eligible studies, is provided in Table 2, according to the types of thyroid autoantibodies examined. For each antibody, the prevalence of antibody positivity in patients with SLE was compared to the prevalence in those without SLE (TgAb: OR = 2.99, 95% CI = 1.83–4.89; TPOAb: OR = 2.20, 95% CI = 1.27–3.82, respectively). The meta-analysis results showed that patients with SLE were more likely to be positive for thyroid autoantibodies than the control groups. Forest plots for the prevalence of TgAb and TPOAb positivity in patients with SLE compared with healthy controls are shown in Fig 2.

**Table 2:** Meta-analysis of the association between SLE and thyroid autoimmunity.

Type of antibody	Eligible studies	OR (95% CI)	*P* value	Heterogeneity test	Effect model
**TgAb**					
Asian	3	5.43 (0.76–39.10)	0.093	P-H = 0.010, *I* ^*2*^ = 78.2%	Random
African	4	2.44 (0.56–10.70)	0.236	P-H = 0.048, *I* ^*2*^ = 62.0%	Random
American	3	4.03 (2.08–7.80)	0.000	P-H = 0.560, *I* ^*2*^ = 0%	Fixed
European	5	1.81 (1.23–2.65)	0.002	P-H = 0.673, *I* ^*2*^ = 0%	Fixed
**Total**	**15**	**2.99 (1.83–4.89)**	**0.000**	**P-H = 0.013, *I*** ^***2***^ = **50.4%**	**Random**
**TPOAb**					
Asian	1	0.66 (0.16–2.76)	0.566	——	Fixed
African	4	4.55 (1.33–15.49)	0.016	P-H = 0.027, *I* ^*2*^ = 67.3%	Random
American	2	1.10 (0.55–2.24)	0.783	P-H = 0.296, *I* ^*2*^ = 8.5%	Fixed
European	4	2.32 (1.62–3.32)	0.000	P-H = 0.185, *I* ^*2*^ = 37.9%	Fixed
**Total**	**11**	**2.20 (1.27–3.82)**	**0.005**	**P-H = 0.003, *I*** ^***2***^ = **62.5%**	**Random**

*TgAb*: thyroglobulin antibody. *TPOAb*: thyroid peroxidase antibody.

Because the included studies examined a geographically diverse set of study populations, a subgroup analysis was performed based on the different continents from which the study subjects were drawn; for this analysis, the subjects in all of the included studies were divided into Asian, African, American, and European populations. The results of the subgroup analysis showed that there was a positive association between TgAb and SLE in both the American and European populations (OR = 4.03, 95% CI = 2.08–7.80; OR = 1.81, 95% CI = 1.23–2.65, respectively), but not in the Asian or African populations (OR = 5.43, 95%CI = 0.76–39.10; OR = 2.44, 95%CI = 0.56–10.70, respectively). A positive association between TPOAb positivity and SLE was observed in the African and European populations (OR = 4.55, 95%CI = 1.33–15.49; OR = 2.32, 95%CI = 1.62–3.32, respectively) but was not observed in either the Asian or the American populations (OR = 0.66, 95% CI = 0.16–2.76; OR = 1.10, 95% CI = 0.55–2.24, respectively). Forest plots showing the results of these subgroup analyses are presented in S1 Fig in the Supporting Information.

### Sensitivity analysis

**Fig 3:**
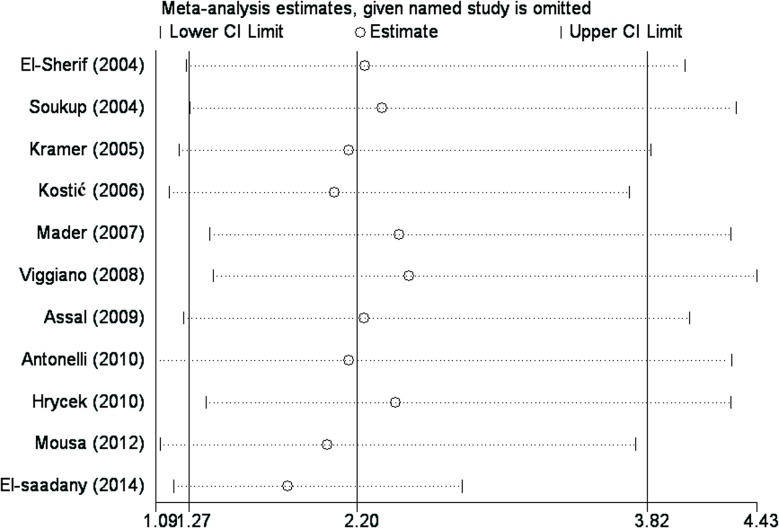
Graphical results of the sensitivity analysis in which the meta-analysis result is re-estimated by omitting each study in turn. For each omitted study listed on the left, summary statistics for the resulting meta-analysis are presented as a horizontal confidence interval. The full, "combined" results are represented by the solid vertical lines.

To investigate the influence of each individual study on the overall meta-analysis estimate, a sensitivity analysis was performed by omitting each study in turn and calculating the pooled OR for the remaining studies sequentially, thus reflecting the influence of each individual data set on the pooled OR. A study was considered to be influential if the pooled OR excluding that study was not within the 95% CI of the overall mean. The ORs resulting from the sensitivity analysis did not differ substantially from our original estimates, indicating that our statistics were relatively stable and credible (Fig 3).

### Publication bias

**Fig 4:**
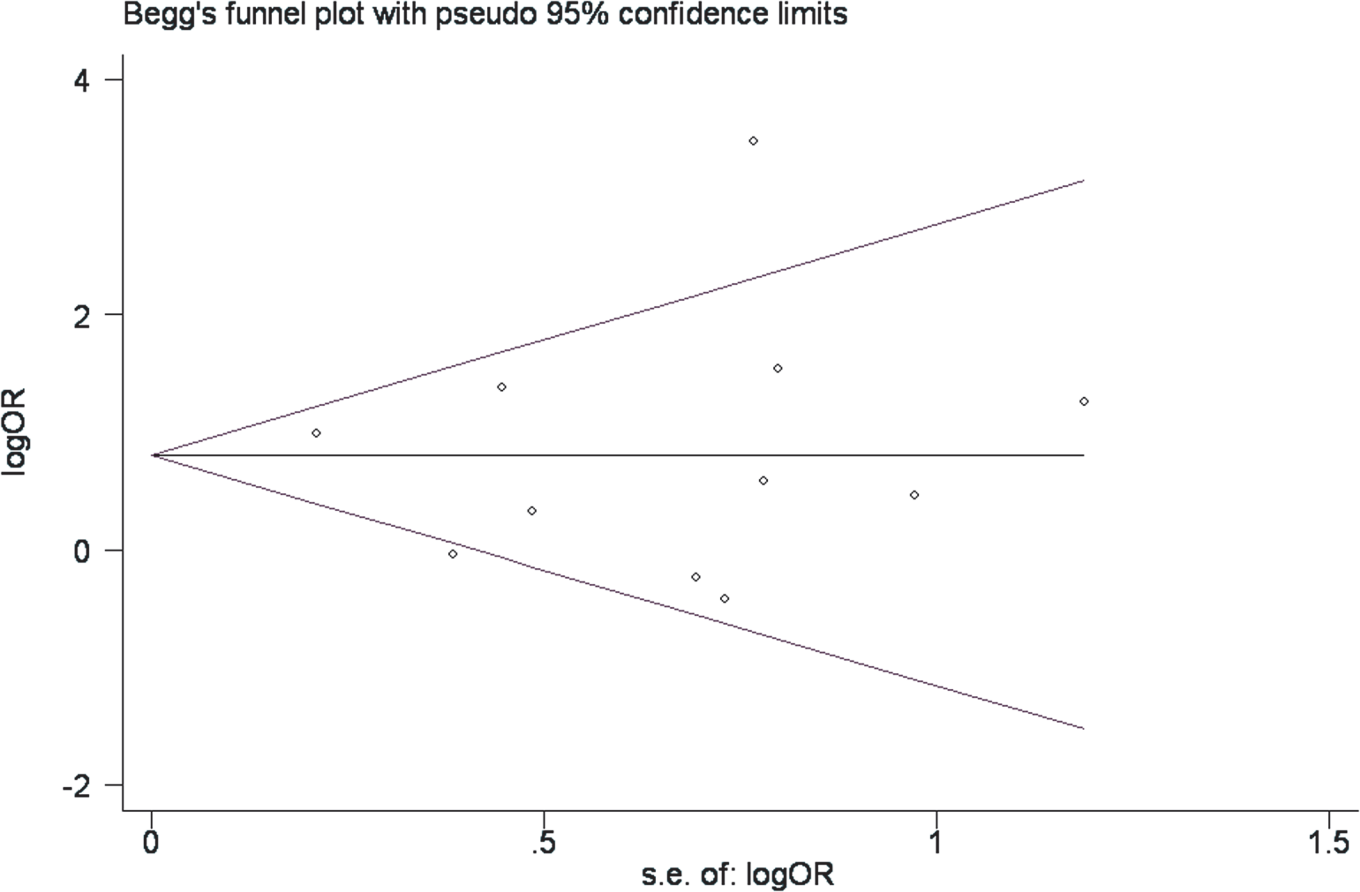
Begg’s funnel plot for testing the publication bias of the association between SLE and the risk of thyroid autoimmunity associated with TPOAb positivity. Each point represents a separate study of the indicated association.

To determine whether a potential publication bias existed in the reviewed literature, a funnel plot was constructed and Begg’s test was conducted. The funnel plot is a relatively straightforward method of determining whether publication bias is present, and Begg’s test was used to examine the symmetries of the plots statistically. The relative symmetry of the distributions indicated that there was no significant publication bias (Fig 4). Similarly, the results of Begg’s test did not suggest the existence of publication bias (TgAb: Pr > |z| = 0.692; TPOAb: Pr > |z| = 0.755, respectively), as all Pr > 0.05.

## Discussion

Thyroid autoimmunity is considered the most common type of organ-specific autoimmune disorder, which can be associated with other autoimmune endocrine problems, as well as non-endocrine diseases [23]. The evaluation of thyroid autoantibody titers is required for the laboratory diagnosis of thyroid autoimmunity. Serum levels of thyroid autoantibodies that exceed or are equal to the upper level of a normal interval represent the minimum criteria for the diagnosis of thyroid autoimmunity. SLE is a prototypical autoimmune disorder with multifactorial etiology [24], which is characterized by chronic inflammation, complement activation, autoantibodies production as well as immune-complex deposition, leading to tissue and organ damage [25]. It is common notion that individuals or family members with autoimmune disease are at higher risk of developing another immune-mediated disorder [26]. Because autoimmune factors may be common features of both thyroid autoimmunity and SLE, it is likely that both diseases may coexist within some patients. The potential association between thyroid autoimmunity and SLE deserves particular attention because it may help us to plan preventive and therapeutic strategies. For example, patients with SLE should pay attention to adjust the environment they lived. Recent studies have indicated that alcohol intake, smoking cessation and iodine intake are all strong factors of risk that, combined, could influence hazard of thyroid autoimmunity [27].

Several studies have evaluated the association between SLE and thyroid autoimmunity, but the results of these studies have been inconsistent. Moreover, most of these studies used a relatively small sample size, which limited their power to detect whether an association between SLE and thyroid autoimmunity is present. Our meta-analysis quantitatively assessed whether an association exists between thyroid autoimmunity and SLE. 15 independent, relevant studies with case-control designs were included in our meta-analysis and assessed. The results of this meta-analysis showed that the prevalence of thyroid autoantibodies in patients with SLE was significantly higher than that in the control groups. The results of a subgroup analysis by geographic location showed that the study subjects with SLE drawn from American and European populations were more likely to be TgAb-positive, but that those drawn from Asian and African populations were not. Regarding TPOAb, there was a positive association between TPOAb positivity and SLE in African and European populations, but this association was observed neither in Asian populations nor in American populations, suggesting a possible role for geography-driven differences in genetic backgrounds and living environments.

The main purpose of performing this meta-analysis was to shed light on the relationship between thyroid autoimmunity and SLE using statistical methods. However, there were some limitations to our meta-analysis. First, several relevant studies were not included in this meta-analysis because of incomplete raw data or other limitations of the publications. Second, in the subgroup analysis, the number of studies examining Asian populations was relatively small; thus, there was not enough statistical power to evaluate the level of association in this group to a desired level of accuracy. Third, due to the lack of consistent criteria for categorizing thyroid function between different countries, this meta-analysis only addresses the relationship between SLE and thyroid autoantibodies. Furthermore, since the studies included were done by different investigators from different countries and at very diverse time points extending for more than 3 decades, the variability of assays used in each study may have some influence to the consequence. Consequently, these meta-analysis results should be interpreted with caution.

In conclusion, the results of our meta-analysis support the hypothesis that the prevalence of thyroid autoantibodies in patients with SLE is higher than that in healthy controls, which suggests that SLE may be associated with increased thyroid autoimmunity risk.

## Supporting Information

S1 PRISMA Checklist (DOC)

S1 Fig Forest plots for the prevalence of TgAb and TPOAb positivity in patients with SLE compared with healthy controls in the subgroup analysis. (TIF)

S1 Table Assessment of the studies’ qualities using the Newcastle-Ottawa Scale. (DOC)

S2 Table The type of assay used by each study included in this meta-analysis, and the cut-off for normality used by each assay. (DOC)
