# Supplementary figures and images for: Patients with Systemic Lupus Erythematosus Have Higher Prevalence of Thyroid Autoantibodies: A Systematic Review and Meta-Analysis
Source: PLoS One. 2015 Apr 23;10(4):e0123291. doi: 10.1371/journal.pone.0123291 (PMC4408090; doi:10.1371/journal.pone.0123291)

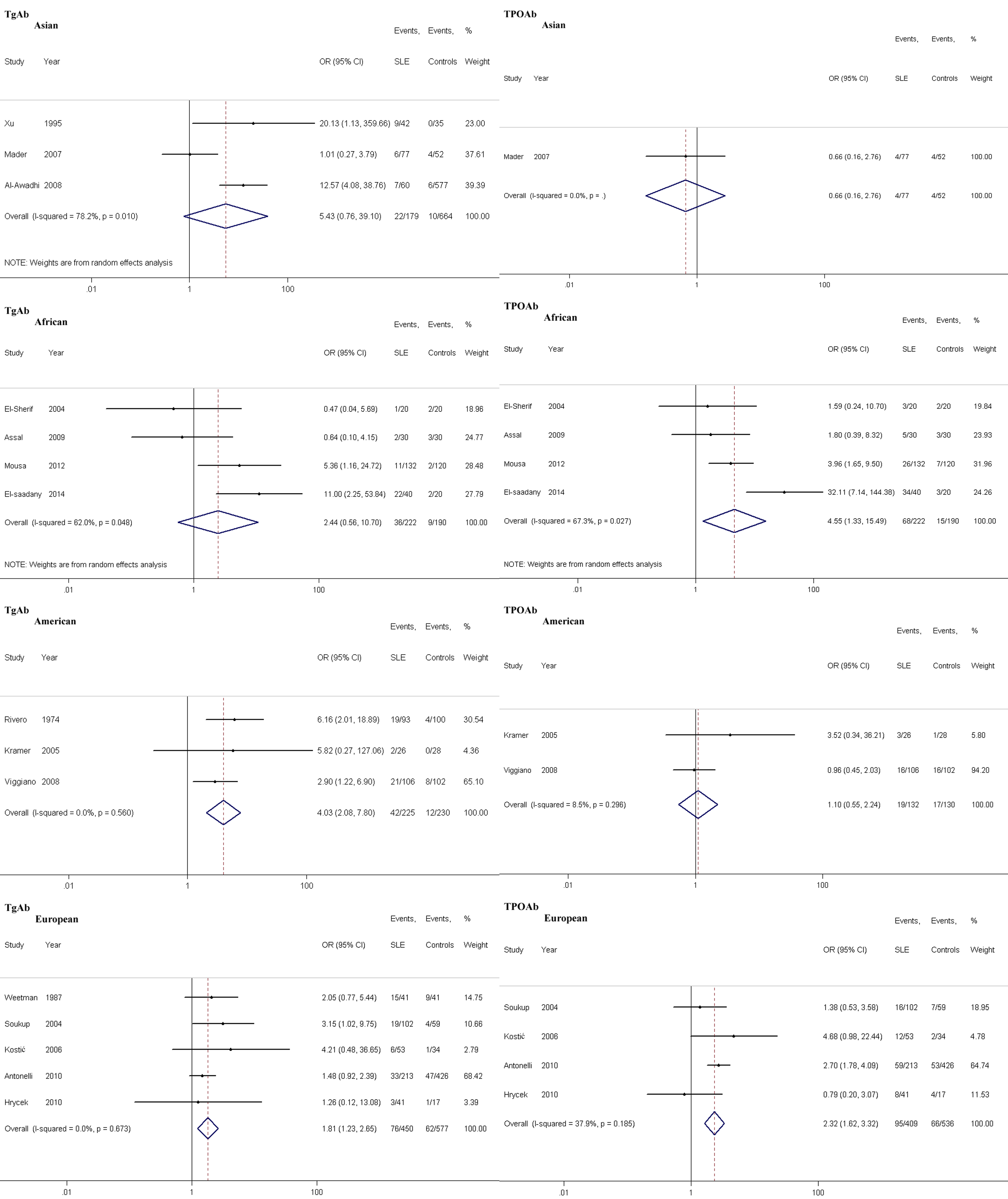

Supplement: S1 Fig — (TIF) [file pone.0123291.s002.tif]
