# Supplementary material for: Patients with Systemic Lupus Erythematosus Have Higher Prevalence of Thyroid Autoantibodies: A Systematic Review and Meta-Analysis
Source: PLoS One. 2015 Apr 23;10(4):e0123291. doi: 10.1371/journal.pone.0123291 (PMC4408090; doi:10.1371/journal.pone.0123291)

**S1 Table. Assessment of the studies’ qualities using the Newcastle-Ottawa Scale.**


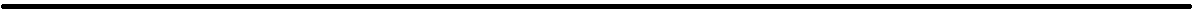


Authors Year Region Selection Comparability Exposure Quality Score


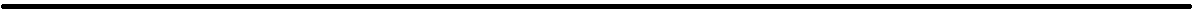


Rivero et al. [8] 1974 Argentina ★★★ ★ ★ ★★★★★

Weetman et al. [9] 1987 UK ★★★ ★★ ★ ★★★★★★

Xu et al. [10] 1995 China ★★ ★ ★★ ★★★★★

El-Sherif et al. [11] 2004 Egypt ★★ ★★ ★★ ★★★★★★

Soukup et al. [12] 2004 Czech ★★★ ★★ ★★★★★

Kramer et al. [13] 2005 Brazil ★★ ★★ ★ ★★★★★

Kostić et al. [14] 2006 Serbia ★★★ ★★ ★★★★★

Mader et al. [15] 2007 Israel ★★ ★ ★★ ★★★★★

Al-Awadhi et al. [16] 2008 Kuwait ★★★★ ★ ★ ★★★★★★

Viggiano et al. [17] 2008 Brazil ★★ ★★ ★ ★★★★★

Assal et al. [18] 2009 Egypt ★★ ★★ ★★ ★★★★★★

Antonelli et al. [19] 2010 Italy ★★★★ ★★ ★ ★★★★★★★

Hrycek et al. [20] 2010 Poland ★★ ★★ ★★ ★★★★★★

Mousa et al. [21] 2012 Egypt ★★★ ★ ★★ ★★★★★★

El-saadany et al. [22] 2014 Egypt ★★ ★★ ★★ ★★★★★★


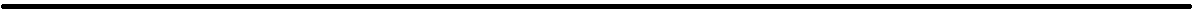


Each study included was judged in three broad categories by using the “star system”: the selection of study groups, the comparability of their cases and controls, and the ascertainment of exposure for cases and controls.


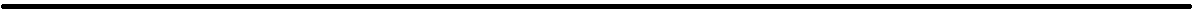

Supplement: S1 Table — (DOC) [file pone.0123291.s003.doc]
