# Supplementary material for: Patients with Systemic Lupus Erythematosus Have Higher Prevalence of Thyroid Autoantibodies: A Systematic Review and Meta-Analysis
Source: PLoS One. 2015 Apr 23;10(4):e0123291. doi: 10.1371/journal.pone.0123291 (PMC4408090; doi:10.1371/journal.pone.0123291)

**S2 Table. The type of assay used by each study included in this meta-analysis, and the cut-off for normality used by each assay.**


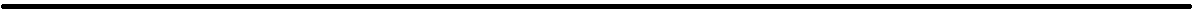


Authors. [Ref] Year Country Continent Comparison Assays Normal cut-off N participants N with thyroid antibodies


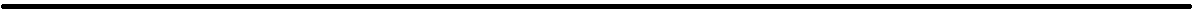


Rivero et al. [8] 1974 Argentina American TgAb passive 1:25 SLE: 93 SLE: 19

hemagglutination Control: 100 Control: 4

Weetman et al. [9] 1987 UK European TgAb ELISA —— SLE: 41 SLE: 15

Control: 41 Control: 9

Xu et al. [10] 1995 China Asian TgAb ELISA —— SLE: 42 SLE: 9

Control: 35 Control: 0

El-Sherif et al. [11] 2004 Egypt African TgAb ELISA 2-50 ng/ml SLE: 20 SLE: 1

Control: 20 Control: 2

TPOAb Immulite analyzer <35 IU/ml SLE: 20 SLE: 3

Control: 20 Control: 2

Soukup et al. [12] 2004 Czech European TgAb ELISA <325 U/ml SLE: 102 SLE: 19

Control: 59 Control: 4

TPOAb ELISA <50 U/ml SLE: 102 SLE: 16

Control: 59 Control: 7

Kramer et al. [13] 2005 Brazil American TgAb agglutination —— SLE: 26 SLE: 2

gel test Control: 28 Control: 0

TPOAb chemoluminescence —— SLE: 26 SLE: 3

Control: 28 Control: 1

Kostić et al. [14] 2006 Serbia European TgAb immunoradiometric <50 U/ml SLE: 53 SLE: 6

assay Control: 34 Control: 1

TPOAb competitive <100 U/ml SLE: 53 SLE: 12

radioligand assay Control: 34 Control: 2

Mader et al. [15] 2007 Israel Asian TgAb immunometric <40 IU/ml SLE: 77 SLE: 6

assay Control: 52 Control: 4

TPOAb immunometric <35 IU/ml SLE: 77 SLE: 4

assay Control: 52 Control: 4

Al-Awadhi et al. [16] 2008 Kuwait Asian TgAb hemagglutination <115 IU/ml SLE: 60 SLE: 7

assay Control: 577 Control: 6

Viggiano et al. [17] 2008 Brazil American TgAb chemiluminescence <50y, <40IU/ml SLE: 106 SLE: 21

>50y, <80IU/ml Control: 102 Control: 8

TPOAb chemiluminescence <50y, <34IU/ml SLE: 106 SLE: 16 >50y, <100IU/ml Control: 102 Control: 16

Assal et al. [18] 2009 Egypt African TgAb ELISA 2-50 ng/ml SLE: 30 SLE: 2

Control: 30 Control: 3

TPOAb immulite <35 IU/ml SLE: 30 SLE: 5

analyzer kit Control: 30 Control: 3

Antonelli et al. [19] 2010 Italy European TgAb immunoradiometric <100 UI/ml SLE: 213 SLE: 33

assay Control: 426 Control: 47

TPOAb immunoradiometric <100 UI/ml SLE: 213 SLE: 59

assay Control: 426 Control: 53

Hrycek et al. [20] 2010 Poland European TgAb immunofluorescence —— SLE: 41 SLE: 3

assay Control: 17 Control: 1

TPOAb immunofluorescence —— SLE: 41 SLE: 8

assay Control: 17 Control: 4

Mousa et al. [21] 2012 Egypt African TgAb ELISA <100 mIu/ml SLE: 132 SLE: 11

Control: 120 Control: 2

TPOAb ELISA <50 mIu/ml SLE: 132 SLE: 26

Control: 120 Control: 7

El-saadany et al. [22] 2014 Egypt African TgAb ELISA —— SLE: 40 SLE: 22

Control: 20 Control: 2

TPOAb ELISA —— SLE: 40 SLE: 34

Control: 20 Control: 3


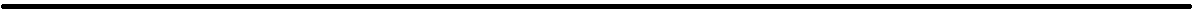


*TgAb*: thyroglobulin antibody. *TPOAb*: thyroid peroxidase antibody.

*n*: number of individuals with positive antibodies. *N*: total number of individuals with SLE disease or healthy controls.


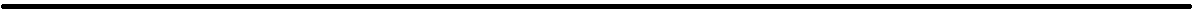

Supplement: S2 Table — (DOC) [file pone.0123291.s004.doc]
